# Parallelizing large networks using NEURON-Python

**DOI:** 10.1186/1471-2202-16-S1-P151

**Published:** 2015-12-18

**Authors:** Alexandra H Seidenstein, Robert A McDougal, Michael L Hines, William W Lytton

**Affiliations:** 1 Dept of Chemical & Biomolecular Engineering, New York University, NY, USA; 2 Dept of Neurobiology, Yale University New Haven CT, USA; 3 Kings County Hospital Center, Brooklyn, NY, USA; 4 Dept. of Physiology & Pharmacology, SUNY Downstate Medical Center, Brooklyn, NY, USA

## 

Research on brain organization has become increasingly dependent on large multiscale neuronal network simulations. Here we describe current usage of the NEURON simulator with MPI (message passing interface) in Python for simulation of networks in the high performance computing (HPC) parallel computing environment [1-4]. The mixed ordinary differential equation (ODE) and event-driven nature of neuronal simulations offers advantages for parallelization, by allowing each node to work independently for a period equivalent to the minimal synaptic delay before exchanging queue information with other nodes, obviating the need to exchange information at every time step.

NEURON's ParallelContext allows access to a few of the important general collective MPI calls, as well as calls adapted from prior usages from the LINDA package, now reimplemented under MPI. From Python, a NEURON ParallelContext is created using pc = h.ParallelContext(), where h provides access to NEURON simulation objects after from neuron import h, ParallelContext permits the periodic transfer of spike information via queue exchanges.

Pseudo-random streams must be consistent regardless of numbers of nodes in order to set connectivity, delays, and weights that are not fully defined from experimental studies. These streams are kept consistent regardless of number of nodes being used and therefore allows for the simulations to be identical. In order to create this, randomizers are established for particular purposes using NEURON's h.Random().Random123().[5] The key to reproducibility is to define each randomizer according to 1. a particular usage, 2. a particular cell (based on a global identifier or gid), 3. a particular run based on a run identifier runid: *e.g.*, after for r in randomizers: r.Random123_globalindex(runid, randomdel.Random123(id32('randomdel'), self.gid, 0) where id32() provides a 32-bit hash for a name: def id32(obj):return hash(obj)&0xffffffff.

Data saving must be initially managed at node of origin and then combined across nodes, to be accessible for analysis or viewing. Given that file saving may occur incrementally during simulation from different nodes on different local filesystems, file management becomes important. There are several ways to handle data saving, the benefits and applicability of which will be presented. Spike recordings are created as vectors on a per-node basis, later consolidated. Other state variables may also be saved at the same time, or may be recreated later utilizing a re-run of individual cells with identical stimulation using NEURON's PatternStim.

We note that ParallelContext in NEURON permits the development of hybrid networks using various types of cells: event-driven cells, integrate-and-fire cells, multicompartment cells, as well as complex cells with calculation of internal chemical milieu. Load balancing in the hybrid circumstance is a crucial issue, particularly when some cells are computationally large due to inclusion of reaction-diffusion mechanisms to develop multiscale models from molecule to network.
